# A novel *PAX6* deletion in a Chinese family with congenital aniridia

**Published:** 2012-04-21

**Authors:** Jian Huan Chen, Weitao Lin, Guoying Sun, Chukai Huang, Yuqiang Huang, Haoyu Chen, Chi Pui Pang, Mingzhi Zhang

**Affiliations:** 1 Joint Shantou International Eye Center, Shantou University & the Chinese University of Hong Kong, Shantou, China; 2 Department of Ophthalmology and Visual Sciences, the Chinese University of Hong Kong, Hong Kong, China

## Abstract

**Purpose:**

To identify a disease-causing paired box 6 (*PAX6*) gene mutation in a Chinese family affected by autosomal dominant congenital aniridia.

**Methods:**

All participants in the study, including the aniridia family and 100 unrelated senile cataract controls, received a comprehensive ophthalmic examination. Genomic DNA was extracted from their whole blood. Mutation screen in all exons and their adjacent splicing junctions of *PAX6* was performed by direct sequencing of polymerase chain reaction (PCR) products. PCR products of heterozygous mutation were further cloned into T-vectors and confirmed by sequencing. Multiple alignments were performed using ClustalX to compare PAX6 protein sequences among vertebrates. MicroRNA binding sites were predicted by TargetScan.

**Results:**

A novel heterozygous *PAX6* deletion c.1251_1353del103 (p.Pro418Serfs*87) affecting exon 14 and the 3′-untranslated-region (3′-UTR) was identified in the congenital aniridia family. The mutation was exclusively observed in all affected family members but not in any unaffected family member or unrelated control. Bioinformatics analysis showed that the deletion led to remarkable changes of the PAX6 protein, including a frameshift, changes of protein sequence, and a COOH-terminal extension. Multiple alignments showed that the affected region of PAX6 shared high sequence identity (100%) among its vertebrate orthologs. The COOH-terminal extension might also affect microRNA binding sites in the 3′-UTR as predicted by TargetScan.

**Conclusions:**

In the current study we reported a novel *PAX6* deletion resulting in an abnormal PAX6 COOH-terminal extension in the Chinese family affected by aniridia. Our findings thus add to the mutation spectrum of *PAX6*.

## Introduction

Congenital aniridia is a severe eye disease that occurs with a prevalence of 1 in 64,000–96,000 across different human populations [1]. Not only the iris but also the lens, optic nerve, cornea, anterior chamber, and retina can be affected by aniridia [2]. About two thirds of aniridia cases are familial, which are autosomal dominant, and the remaining one third are sporadic [3].

The majority of mutations in congenital aniridia have been found in the paired box 6 gene (*PAX6*) [4]. The gene is a member of the paired box gene family. Encoding a transcription factor, *PAX6* spans more than 22 kb at chromosome 11p13, contains 14 exons and generates 3 isoforms of transcripts by alternative splicing [5]. The PAX6 protein consists of 3 domains as follows: a paired domain (PD) in the NH_2_-terminus, a proline-serine-threonine domain (PST) in the COOH-terminus and a homeodomain (HD) in-between [6]. At the present time, the total number of mutations reported is more than 700 [4]. Most of *PAX6* mutations are found in exons 5–14, causing various severe phenotypes in the eye such as aniridia [3,5], cataract [7] and possibly myopia [8-10], all of which could eventually lead to blindness.

Although *PAX6* mutations have been reported in Chinese [11-27], disease phenotypes vary among different *PAX6* mutations. The phenotype-genotype correlation, which is important in the understanding of the disease mechanism, remains to be further elucidated. In the current study, we screened all exons and their adjacent splicing junctions in *PAX6* in a Chinese family with severe disease phenotypes including congenital aniridia and myopia, and identified a novel 103 bp deletion, which caused a frameshift and an abnormal COOH-terminal extension at the protein level.

## Methods

### Subject recruitment and clinical examination

**Figure 1:**
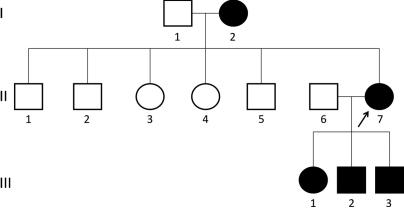
Pedigree of a Chinese family with aniridia. Filled squares and circles denote affected males and females, respectively. Normal individuals are shown as empty symbols. The proband is indicated by an arrow.

The family with congenital aniridia was recruited at the Joint Shantou International Eye Center, Shantou, China (Figure 1). One-hundred senile cataract controls without aniridia were recruited from surgical inpatients at the Hospital. Visual acuity, refraction error, intraocular pressure, and slit lamp examinations were performed and documented in all participants (clinical details of individual I-2 could not be obtained due to her old age). Spectral-domain optical coherence tomography (Cirrus HD-OCT; Carl Zeiss Meditec, Inc., Berlin, Germany) was conducted for individual III-2. Color Doppler ultrasound examination of the kidney for detection of Wilms’ tumor was performed at the Chaonan People Hospital for affected family members. Peripheral blood was collected from 6 members of the family (II-1, II-6, II-7, III-1, III-2, and III-3) and all senile cataract controls. Genomic DNA was extracted by using the QIAmp Blood kit (Qiagen, Hilden, Germany).

This study was approved by the Ethics Committee of Joint Shantou International Eye Center and was conducted in accordance with the Declaration of Helsinki. Written consent was obtained from each participating subject after explanation of the nature of the study.

### Mutation screening

Thirteen pairs of primers (Table 1) for amplicons targeting all exons and their adjacent splicing junctions in *PAX6* (NCBI human genome build 37.2, NC_000011 for gDNA, NM_001604, NP_001595), were modified from a previously study [21]. The polymerase chain reaction (PCR) amplification was performed using the GeneAmp PCR System 9700 (ABI, Foster City, CA) in a 25-µl mixture containing 1.5 mM MgCl_2_, 0.2 mM of each dNTP (Sangon, Shanghai, China), 1 U Taq DNA polymerase (Invitrogen, Carlsbad, CA), 0.2 µM primers and 20 ng of genomic DNA. Bidirectional sequencing of PCR products was performed using the BigDye Terminator Cycle Sequencing v3.1 kit (ABI) and the 3130xl Genetic Analyzer (ABI). Haplotyping was used to confirm the mutation sequence. PCR products of heterozygous mutants were ligated to pMD^®^18-T vectors (Takara, Dalian, China), and subsequently sequenced by the 3130xl Genetic Analyzer. Mutation naming followed the nomenclature recommended by the Human Genomic Variation Society (HGVS).

**Table 1:** Primers used for PCR and sequencing of *PAX6*.

**Target**	**Exon ID**	**Primer sequence (5′-3′)**	**Annealing temperature (°C)**	**Product length (bp)**
1a*	E1aF	AGCTGTGCCCAACTCTAGCC	57	399
** **	E1aR	TTCCATCTTTGTATGCCTCCTT	** **	** **
1	E1F	CGGAGCCGAAAACAAGTG	57	388
** **	E1R	GAGTGTGGGTGAGGGAAGTG	** **	** **
2	E2F	CCACTTCCCTCACCCACAC	60	422
** **	E2R	CTCCTGCGTGGAAACTTCTC	** **	** **
3	E3F	AAGTGGGATCCGAACTTGC	57	349
** **	E3R	CAGCCACCACAGAACTTGC	** **	** **
4	E4F	CAAGCCCCAAAGGGTAGATT	57	286
** **	E4R	CGAAGTCCCAGAAAGACCAG	** **	** **
5	E5F	GGCTGGTGGTCCTGTTGTCCTT	58	441
** **	E5R	GAGGGCGTTGAGAGTGGAG	** **	** **
6,7	E6–7F	AAGCAAGGTCAGCACAAAAATAAATT	64	648
** **	E6–7R	GGAGGAGGTAAAGAGGAGAGAGCATT	** **	** **
8	E8F	TAAGGTTGTGGGTGAGCTGAGATG	66	315
** **	E8R	GGGAGAGTAGGGGACAGGCAAAGG	** **	** **
9	E9F	TTTGGTGAGGCTGTCGGGATATAAT	58	415
** **	E9R	TGCCCAGAGAAATAAAAAGACAGAAA	** **	** **
10	E10F	TTGGTTGGAGGTAATGGGAGTGG	61	334
** **	E10R	TGGCAGCAGAGCATTTAGCAGAC	** **	** **
11,12	E11–12F	GGGGCTGGGCTCGACGTAG	62	438
** **	E11–12R	GCCACCACCAGCCGCACTTA	** **	** **
13	E13F	GGGGCTGTGGCTGTGTGATGT	61	333
** **	E13R	CCCCAGGGACAAGGAAAGCAA	** **	** **
14	E14F	CCAAACATGCAAACAAACAGAGGA	52	570
** **	E14R	TTCCAACTGATATCGTGCCTTCTG	** **	** **

*: This extra exon is located at 5′ upstream of exon1, existing in another alternatively spliced transcript variant NM_001127612.1 but not in NM_001604.4.

### Bioinformatics analysis

Sequences of PAX6 orthologs in other vertebrate species were retrieved from the NCBI Reference Sequence database. Multiple alignment of PAX6 orthologs from different vertebrate species was conducted using ClustalX version 2.0 [28]. MicroRNA targets in the *PAX6* 3′-UTR was predicted by TargetScan release 6.0 [29].

## Results

### Clinical data

**Figure 2:**
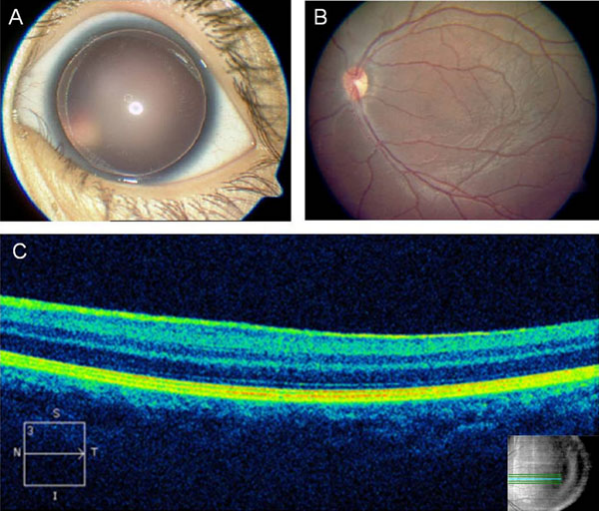
Photos and images showing the clinical features of affected patients. **A**: Complete absence of iris in III-2 (OS). **B**: Foveal hypoplasia observed in III-2 (OS). **C**: A flat fovea in III-2 (OS) demonstrated by optical coherence tomography.

As illustrated by Table 2 and Figure 2, four affected patients (II-7, III-1, III-2, and III-3) receiving ophthalmic examination showed similar clinical symptoms, including congenital aniridia, foveal hypoplasia, and nystagmus (Figure 2). Intraocular hypertension without evident glaucoma was also observed (except the youngest child, III-3). Other clinical features were characterized by normal corneas and transparent lens. The proband (II-7) had low visual acuity, and had high myopia with refractive errors equal to −18 diopters (OD) and −12 diopters (OS). Highly myopic status indicated by relative elongated eye axial length was found in the eldest child (III-1; OD: 22.82 mm; OS: 24.10 mm) with regards to his age of 6 years. Myopia was not observed in the other two children (III-2 and III-3) probably due to their younger ages. Color Doppler ultrasound examination showed that there was no Wilms’ tumor in the four affected patients.

**Table 2:** Demographic and clinical data of the aniridia family.

** **	** **	** **	**Ophthalmic anomalies**						** **
Subject*	**Age (years)**	**Gender**	**Complete aniridia**	**Foveal hypoplasia**	**Nystagmus**	**High myopia**	**Congenital cataract**	**Ocular Hypertension**	**Wilms’ tumor**
I-2	71	F	+	NA	NA	NA	-	NA	NA
II-7	31	F	+	+	+	+	-	+	-
III-1	6	F	+	+	+	+	-	+	NA
III-2	5	M	+	+	+	-	-	+	-
III-3	2	M	+	+	+	NA	-	-	-

M: male; F: female; NA: not available; * All of the other families members have complete iris, without ocular hypertension or high myopia or other major eye diseases, and thus are not listed the table.

### Mutation analysis

**Figure 3:**
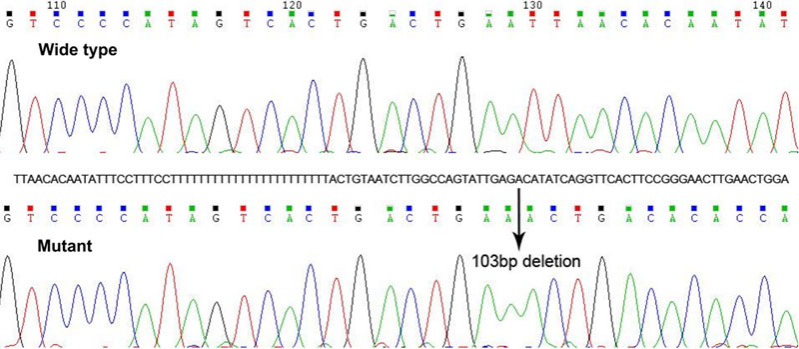
Confirmation of the *PAX6* deletion in the aniridia family by direct-sequencing. The sequence in the plus strand of chromosome 11 is shown. The upper panel is the chromatogram of the *PAX6* wild type, and the lower panel is the chromatogram of the mutant. The arrow indicates the position of the deletion, and the deleted sequence is shown above the arrow.

**Figure 4:**
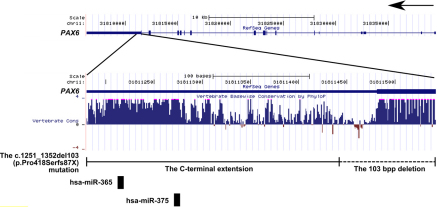
Diagram of the human *PAX6* gene and the deletion with the consequent COOH-terminal extension in the current aniridia family. MicroRNA targets are predicted by TargetScan.

**Figure 5:**
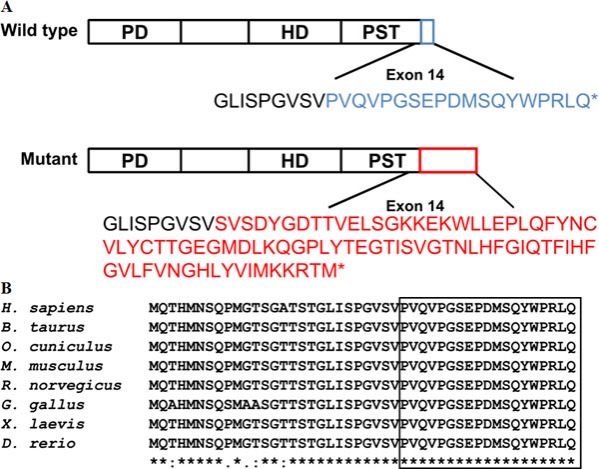
The deleted region in the PAX6 protein is highly conserved among vertebrates. **A**: Diagrams of the wild type and mutant PAX6 proteins. Upper panel represents a human wild type PAX6 protein. The Lower panel represents a mutant. Due to the frameshift and COOH-terminal extension generated by the DNA deletion, the peptide in blue encoded by exon 14 of the wild type is replaced by the peptide in red in the mutant. **B**: Multiple alignment of PAX6 COOH-terminal sequences from different species of vertebrates. The box indicated that the deleted peptide shares 100% identity among vertebrates.

By the means of direct sequencing and haplotyping, a heterozygous 103 bp deletion (c.1251_1353del103, p.Pro418Serfs*87) was identified in all affected family members, which consisted of part of exon 14 and 3′-UTR (Figure 3 and Figure 4). The mutation was not found in any unaffected family member or senile cataract control. No other mutation was detected from any other *PAX6* exon in the affected family members. The mutation caused a frameshift and was predicted to generate proteins with an abnormal COOH-terminal extension, in which 19 amino acid residues starting from codon 418 were replaced by a peptide of 86 amino acid residues (Figure 5A). Multiple alignments of PAX6 sequences from different vertebrate species revealed 100% identity of the deleted region at PAX6 COOH-terminus, which suggested that it was highly conserved during evolution (Figure 5B). TargetScan analysis predicted a target site of hsa-miR-365 and another target site of has-miR-375 at the wild type *PAX6* 3′-UTR, which was converted into coding region by the shifted open reading frame in the mutant (Figure 4).

## Discussion

In the current study with a family affected by aniridia, we identified a novel 103 bp deletion (c.1251_1353del103, p.Pro418Serfs*87), which generated a frameshift and a COOH-terminal extension. The deletion was correlated with aniridia disease phenotypes in an autosomal dominant pattern, but did not affect the cornea or lens. These findings suggested that COOH-terminal extension mutations of *PAX6* could also cause severe aniridia phenotype in the Chinese population, which was comparable to those reported in Caucasians [10].

In Caucasians it was reported that the most common *PAX6* mutations were premature termination mutations, amino acid substitutions and COOH-terminal extensions [10]. There were only limited data of *PAX6* COOH-terminal extension mutations reported in Chinese [25]. Therefore, COOH-terminal extensions of *PAX6* in Chinese remained to be further investigated. The *PAX6* deletion found in our study (c.1251_1353del103) led to a frameshift, and caused a COOH-terminal extension into the 3′-UTR (p.Pro418Serfs*87). It also changed the COOH-terminal amino acid sequence by replacing the last 19 amino acid residues with a peptide of 86 amino acid residues, and possibly affected the PST domain of PAX6 (Figure 5). Sequence analysis showed that the deleted residues in the PST domain were highly conserved among different vertebrate species from Zebrafish to humans (Figure 5), implicating its substantial role in ocular and neurologic development. The PST domain is a transactivation domain and participates in modulating the DNA binding function of PAX6 [30-33]. Brain-expressed proteins interact with PAX6 through the COOH-terminus and with the entire PST domain [34]. The COOH-terminus extension in our Chinese family possibly disrupted the function of PST domain, and could cause severe ocular anomalies including aniridia in an autosomal dominant pattern.

Phenotypic consequence in different types of *PAX6* mutations was reported in Chinese aniridia patients [11-27], but the understanding of that in COOH-terminal extension mutations remained to be further studied. Previously Zhang et al. [25] reported a COOH-terminal extension mutation c.1268A>T (X423LeuextX*15) in aniridia patients, but the mutation was also found in a patient with cataract and intact iris, probably due to incomplete penetrance. In Caucasians the severity of anomalies in COOH-terminal extension mutations were comparable to that in premature termination codon mutations, including iris anomalies, foveal hypoplasia, keratopathy, cataracts and myopia [9,10]. Intriguingly, in our Chinese aniridia family the mutation had part of the last exon and the 3′-UTR in *PAX6* deleted, and resulted in a COOH-terminal extension, probably without triggering nonsense-mediated mRNA decay [35]. Among the patients bearing the mutation, the anomalies included aniridia, intraocular hypertension and high myopia, although myopic status remained to be confirmed in the two younger children due to their young ages. If untreated, glaucomatous damage might also develop in these affected patients. Taken together, these findings possibly were in line with a suggestive role played by the *PAX6* 3′-UTR in myopia etiology. Single nucleotide polymorphisms in the *PAX6* 3′-UTR were associated with myopia in Chinese [36,37]. The *PAX6* 3′-UTR might be involved in myopia development by regulating the expression of *PAX6* mRNAs, which was similar to short repeats in *PAX6* promoter [8]. Analysis by TargetScan predicted targets of microRNAs hsa-miR-365 and has-miR-375 encompassed by the COOH-terminal extension (Figure 4). To date, there has been no report directly studying the role of microRNA targeting *PAX6* in aniridia. Nevertheless, microRNAs targeting other genes in the *PAX6* pathway have been reported to be involved in eye development. For example, Meis homeobox 2 (*MEIS2*) modulates *PAX6* transcription activity, and in madaka fish morpholino-mediated ablation of miR-204 targeting *Meis2* results in an eye phenotype characterized by microphthalmia, abnormal lens formation, and altered dorsoventral patterning of the retina [38]. Therefore, the conversion from microRNA binding sites at the wild type *PAX6* 3′-UTR to coding region in the mutant, may abolish the regulation capacity of has-miR-365 and has-miR-375, and further elicit increased *PAX6* mRNA expression, and consequently aniridia and high myopia. In addition, our data showed that the mutation did not cause either anomalies in the cornea or lens, or systemic symptoms in the kidney such as Wilms’ tumor. Our genotype-phenotype analysis thus provided useful information for understanding functional consequences of COOH-terminal extension mutations in *PAX6*.

In the present study, we found a novel *PAX6* deletion in a Chines family affected by congenital aniridia. Our findings thus expanded the mutation spectrum of *PAX6* gene in Chinese.
